# Anti-inflammatory effects of α-MSH through p-CREB expression in sarcoidosis like granuloma model

**DOI:** 10.1038/s41598-020-64305-9

**Published:** 2020-04-29

**Authors:** Chongxu Zhang, Stephanie Chery, Aaron Lazerson, Norman H Altman, Robert Jackson, Greg Holt, Michael Campos, Andrew V Schally, Mehdi Mirsaeidi

**Affiliations:** 1 Section of Pulmonary, Miami VA Health System, Miami, FL USA; 2 Departments of Medicine University of Miami Miller School of Medicine, Miami, FL USA; 3 Comparative Pathology, University of Miami Miller School of Medicine, Miami, FL USA; 4 Division of Pulmonary and Critical Care, University of Miami Miller School of Medicine, Miami, FL USA; 5 Polypeptide and Cancer Institute, Veterans Affairs Medical Center, Miami, FL USA

**Keywords:** Experimental models of disease, Molecular medicine

## Abstract

Lung inflammation due to sarcoidosis is characterized by a complex cascade of immunopathologic events, including leukocyte recruitment and granuloma formation. α-melanocyte stimulating hormone (α-MSH) is a melanocortin signaling peptide with anti-inflammatory properties. We aimed to evaluate the effects of *α*-MSH in a novel *in vitro* sarcoidosis model. An *in vitro* sarcoidosis-like granuloma model was developed by challenging peripheral blood mononuclear cells (PBMCs) derived from patients with confirmed treatment-naïve sarcoidosis with microparticles generated from *Mycobacterium abscessus* cell walls. Unchallenged PBMCsand developed granulomas were treated daily with 10 μM *α*-MSH or saline as control. Cytokine concentrations in supernatants of culture and in cell extracts were measured using Illumina multiplex Elisa and western blot, respectively. Gene expression was analyzed using RNA-Seq and RT-PCR. Protein secretion and gene expression of IL-7, IL-7R, IFN-*γ*, MC1R, NF-κB, phosphorylated NF-κB (p-NF-κB), MARCO, and p-CREB were measured with western blot and RNAseq. A significant increase in IL-7, IL-7R, and IFN-*γ* protein expression was found in developed granulomas comparing to microparticle unchallenged PBMCs. IL-7, IL-7R, and IFN-*γ* protein expression was significantly reduced in developed granulomas after exposure to *α*-MSH compared with saline treated granulomas. Compared with microparticle unchallenged PBMCs, total NF-κB and p-NF-κB were significantly increased in developed granulomas, while expression of p-CREB was not changed. Treatment with *α*-MSH promoted a significantly higher concentration of p-CREB in granulomas. The anti-inflammatory effects of *α*-MSH were blocked by specific p-CREB inhibition. *α*-MSH has anti-inflammatory properties in this *in vitro* granuloma model, which is an effect mediated by induction of phosphorylation of CREB.

## Introduction

Sarcoidosis is a multi-organ granulomatous disease of unknown etiology that affects thousands of people around the world and is associated with significant morbidity and mortality^1^. In affected organs, sarcoidosis triggers an inflammatory reaction characterized by cellular recruitment of type 1 T helper cells (Th1) followed by macrophages that play a crucial role leading to granuloma formation^2^. The etiology and pathogenesis of sarcoidosis is poorly understood, which has limited the development of an effective *in vitro* sarcoidosis model. Bacterial antigens are shown to be associated with sarcoidosis. mycobacterial proteins are the most common antigens isolated from sarcoidosis lesions of the lungs^3–6^. In addition, antigen-specific immune responses to mycobacterial virulence factors have been detected in bronchoalveolar lavage fluid (BALF) from sarcoidosis patients^7^. These data suggest that at least in some patients, sarcoidosis may occur due to abnormal inflammation in response to mycobacterial antigens.

Approximately 50% of sarcoidosis patients require systemic steroid therapy. However, up to 20% of treated patients continue to exhibit a persistent granulomatous inflammatory process with progression to tissue remodeling and fibrosis^8^. The US Food and Drug Administration (FDA) has approved only two medications to treat sarcoidosis: prednisone and repository corticotropin injections (approved in 1952)^9,10^. The persistence of symptoms and the involvement of vital organs demand prolonged treatment courses, often associated with additional comorbidities. For this reason, alternative less toxic therapeutic agents with equal or higher efficacy are urgently needed.

Recently, we reviewed the role of α-Melanocyte-stimulating hormone (α-MSH) in reducing inflammation^11^. α-MSH is a 13-amino acid peptide produced by post-translational processing of the hormone proopiomelanocortin (POMC), which has been shown to have anti-inflammatory properties in ocular and intestinal tissues^12–14^. α-MSH activates its receptor, Melanocortin 1 (MC1R), which in return acts downstream via JAK-STAT pathway to activate cAMP response element-binding protein (CREB). CREB is a transcription factor which binds to DNA and increases expression of anti-inflammatory genes^11^.

In this study, we explored the anti-inflammatory properties of α-MSH by measuring the expression of phosphorylated CREB (p-CREB) in a granuloma before and after exposure to α-MSH. We developed *in vitro* granuloma by exposing human peripheral blood mononuclear cells (PBMCs) to microparticles generated from mycobacterial cell walls. This granuloma was immunophenotypically similar to a sarcoidosis granuloma with dominant Th1 and Th17 responses.

## Results

### Development of an In vitro granuloma model

Given the association between mycobacteria and sarcoidosis^15–17^, we developed microparticles from *Mycobacterium abscessus* (MAB) cell wall to stimulate T cells and monocytes from PBMCs to develop granulomas. MAB cell wall microparticles were isolated as described. 8 strains of MAB with a rough colony isolated from patients (were gifted by Dr. Malin Ridell, University of Gothenburg, Sweden) and 2 strains isolated from the environment (soil samples) were used. The size of the particles was measured by analyzing high-quality scanning electron microscope (SEM) images, ranging from less than a sub-micron to 2μm (shown in Figure S1). To prove that the microparticles were bacteria free, they were cultured to confirm no growth before each experiment.

**Figure 1:**
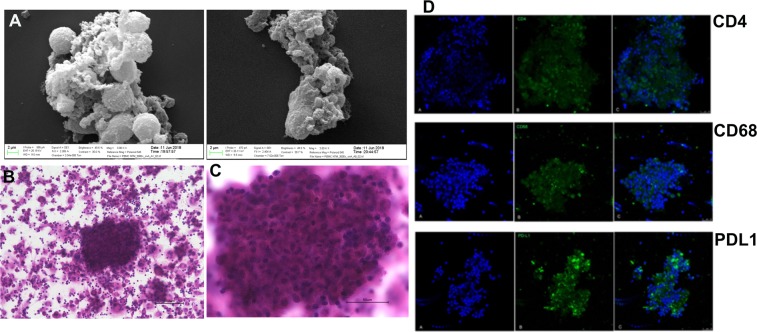
Shows developed *in vitro* granuloma from PBMC of subjects with sarcoidosis. A shows SEM images of granuloma including cells aggregations and confluent cells. B shows granuloma with X20 and C shows granuloma with 60X magnifications. The granuloma sized more than 150 microns as shown in C. IHC from matured granuloma developed *in vitro* from PBMC of subjects with sarcoidosis. Top row shows CD4, middle shows CD68, and bottom row shows PD-L1 expressions in granuloma. PD-L1 shows activation of probable T cells and macrophages.

MAB particles <2μm with an equivalent multiplicity of infection (MOI) of 10:1 (and a total endotoxin level of ≤ 1.115 EU/ml) were incubated with PMBCs extracted from treatment-naive individuals with sarcoidosis who had negative tuberculosis IFN-γ release assays (IGRA). After 72 h, H&E staining and SEM imaging of cultures revealed cellular structures consistent with matured granulomas. Granuloma features were confirmed via immunohistochemistry by showing positive stains for CD4^+^, CD68^+^ as well as PD-L1 as shown in Fig. 1.

### **Characterization of the*in vitro*granuloma model T-helper immunophenotype**

#### Gene expression profile

Given that granulomas from the subjects with clinical sarcoidosis have Th1 and Th17 gene expression profiles^18^, we tested whether this occurred as well in the *in vitro* granuloma model. For this, we analyzed the induced granulomas for gene expression using RNAseq. We found that the *in vitro* granulomas had 853 genes which were significantly differentially expressed (FDR < 0.05 and FC > 2.5) compared to unchallenged PBMCs. These genes included IL-1β, IL-2R, IL-6, IL-7, IL-8, IL-10, IL-12, IL-15, IFN-𝛼, IFN-γ, TNF-𝛼, GM-CSF, CCL2, CCL3, CCL4, CCL5, CXCL9 and CCL11. Pathway analysis performed on the differentially expressed genes was done using iPAthwayGuide software (ADVAITA, Plymouth, MI) and confirmed a gene expression profile enriched for Th1 and Th17 pathways^19^. More information could be found in the supplement document (Figures S2, S3 and S4**)**.

#### Cytokine profile

**Figure 2:**
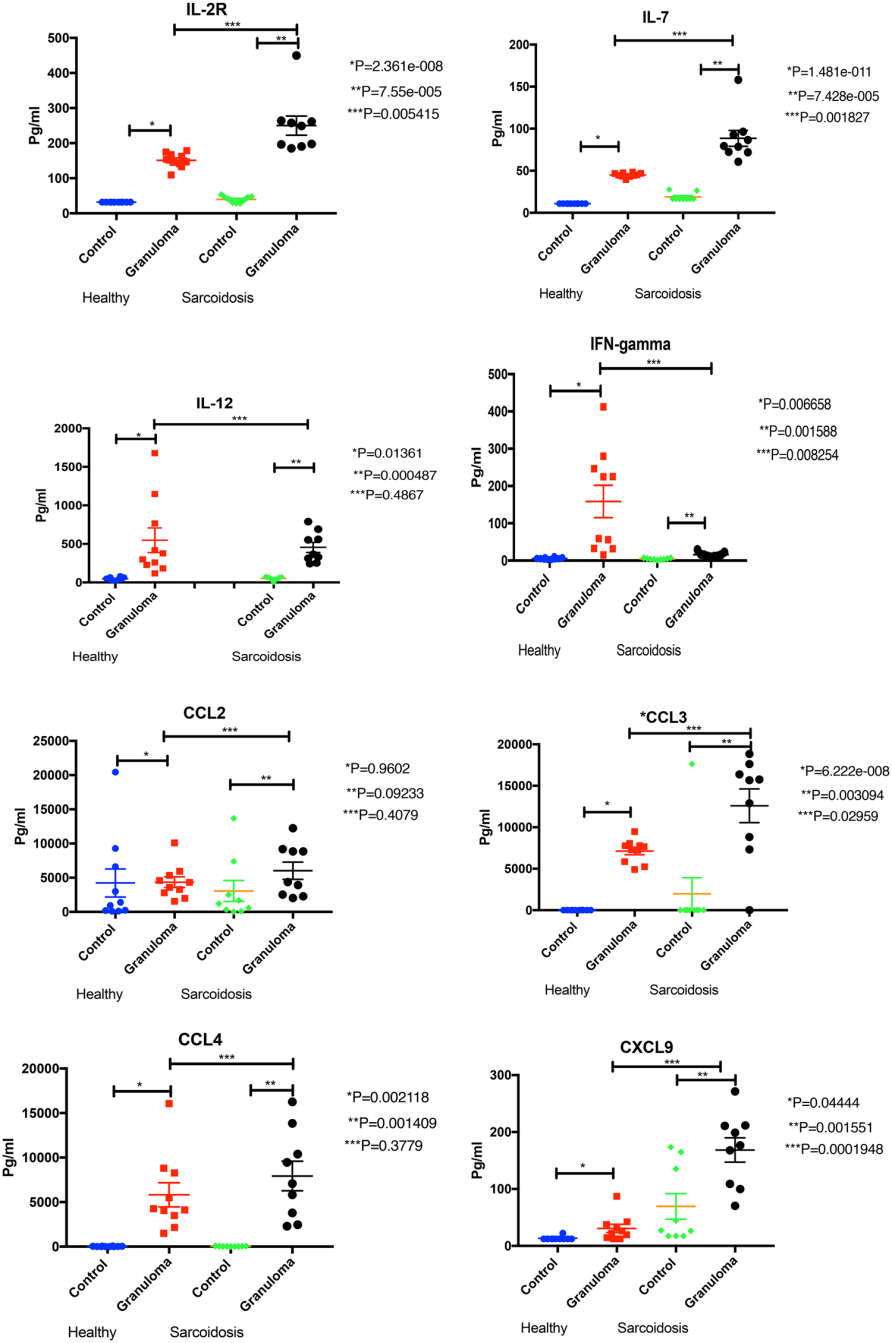
Shows cytokines concentrations of *in vitro* granulomas compared to unchallenged PMBC isolated from healthy control and sarcoidosis subjects. The secretion of Th1 cytokines (IL-2R, IL7, IL-12, and IFN-gamma), macrophage related cytokines (CCL2, CCL3, CCL4, CXCL9), other cytokines (TNF-alpha, GM-CSF, IL-6, IL-10, CCL5 and CXCL10) in granuloma developed from PBMC of sarcoidosis subjects and healthy controls.

We also evaluated the cytokine release profile of the *in vitro* granuloma model since granulomas in clinical sarcoidosis release cytokines with a Th1 profile^20,21^. Supernatants of formed granulomas were collected on Day 3 and analyzed for IL-2R, IL-6, IL-7, IL-10, IL-12, IFN-*γ*, TNF-*𝛼*, GM-CSF, CCL2, CCL3, CCL4, CCL5, CXCL9, CXCL10 and CCL11 cytokine concentrations using Illumina multiplex ELISA according to the manufacturer’s recommendations. Figure 2 shows that *in vitro* granulomas released a significantly higher concentration of cytokines compared to equivalent number of unchallenged PMBCs. The granuloma model demonstrated a Th1 cytokine profile with increased secretion of IL-2R, IL-7, IL-12, IFN-*γ*, and TNF-*𝛼*.

Figure 2 also shows that the cytokine release profile was more pronounced in granulomas derived from PBMCs from sarcoidosis subjects, as opposed to healthy controls. Controls were age, gender and race matched to the patients with treatment-naïve sarcoidosis. Overall, the MAB-induced granulomas from sarcoidosis patients expressed higher concentrations of IL-6, IL-7, IL-2R, CCL2, CCL3, CCL4, CXCL9, and CXCL10 but less IL-12, CCL5 and IFN-*γ*. Of particular note, the granulomas from sarcoidosis patients had a marked decrease in IFN-*γ* expression compared to the healthy controls. PBMCs from healthy subjects with a prior exposure to mycobacterial antigens, as defined by having a positive PPD, did not show any change in their cytokine release profile (Figs. S6a and 6b). These data support the notion that sarcoidosis patients have a distinct response to mycobacterial components. This finding may shed light on pathogenesis of sarcoidosis.

#### NF-κB expression

**Figure 3:**
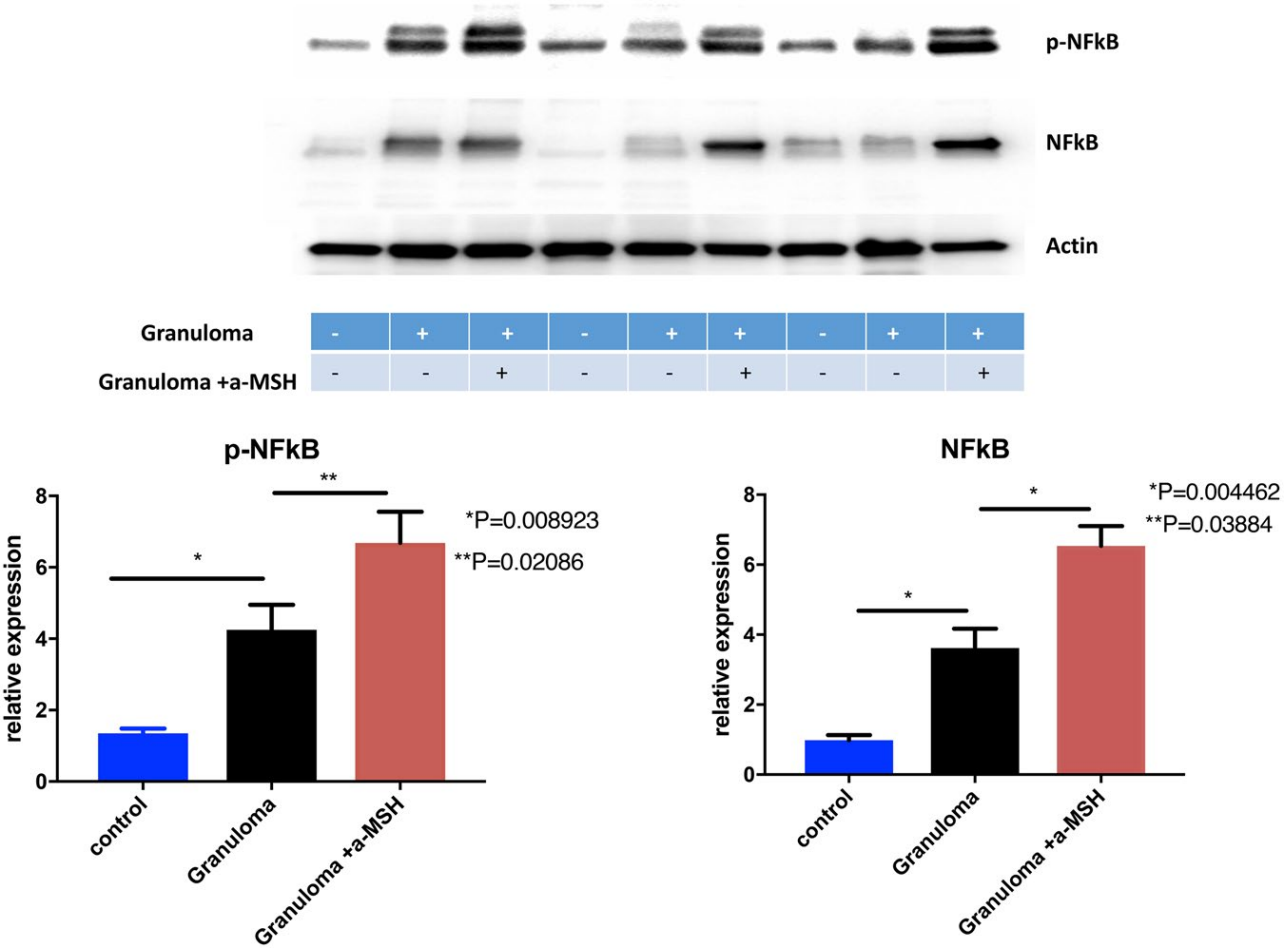
Shows NF-κB and p-NF-κBconcentration increased in granuloma and increased further after treatment with a-MSH. Granuloma was developed using PBMC of sarcoidosis subjects for this experiment.

**Figure 4:**
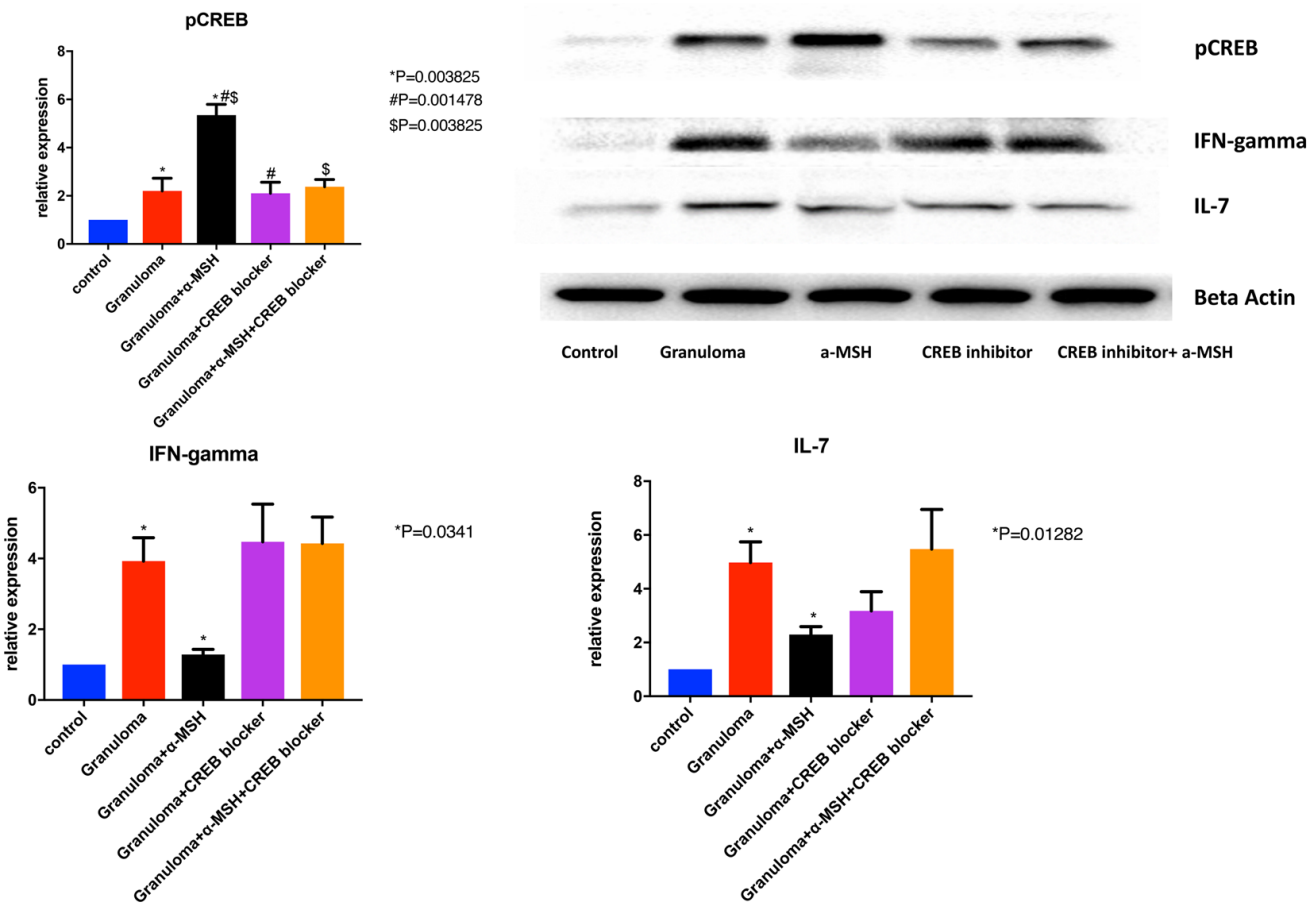
Shows that phosphorylated CREB concentration increase in granuloma. This effect was reversible with adding CREB blocker. This figure also shows that expression of IFN-gamma and IL-7 increased in granuloma and significantly reduced after treatment with α-MSH. Granuloma was developed using PBMC of sarcoidosis subjects for this experiment. + and − in the figure show which one is granuloma (+ yes and −no), and treated with α-MSH (+yes, and −no).

The heightened T-cell immunophenotype of the *in vitro* granulomas was associated with a significantly higher protein expression for total NF-κB and phosphorylated NF-κB (p-NF-κB), compared to unchallenged PBMCs from subjects with sarcoidosis while the p-CREB did not have any statistically significant change in *in vitro* granulomas as shown in Figs. 3 and 4. This suggests that p-NF-κB plays a central role in the induction of inflammation after exposure to microparticles.

### α-MSH exerts anti-inflammatory effects on MAB-induced *in vitro* granulomas

To evaluate the potential anti-inflammatory effects of 𝛼-MSH in sarcoidosis inflammation, we tested its effects on the *in vitro* granuloma model using PBMCs from subjects with sarcoidosis exposed to MAB particles. We performed RNA-Seq analysis on day 3 after exposure to 10uM 𝛼-MSH. Figure S7 shows genes that were differentially expressed in granuloma treated with 𝛼-MSH compared to controls with granuloma not treated with 𝛼-MSH. Figure S8 shows biologic processes of 21 genes that significantly were up- or down-regulated after 𝛼-MSH treatment in granulomas. We performed RT-PCR analysis at day 3 after exposure to 10uM 𝛼-MSH. We observed that RNA expression of *IL-7*, *IL17A*, *IL6*, *MARCO*, *IFN-*γ, and *IL-8* was significantly decreased in granulomas treated with α-MSH compared to the controls (Figure S9). RNA-seq analysis showed significant changes in gene profiles between the two groups as shown in heatmap (see Figure S10). No change in the granuloma size was noted after treatment with 𝛼-MSH.

**Figure 5:**
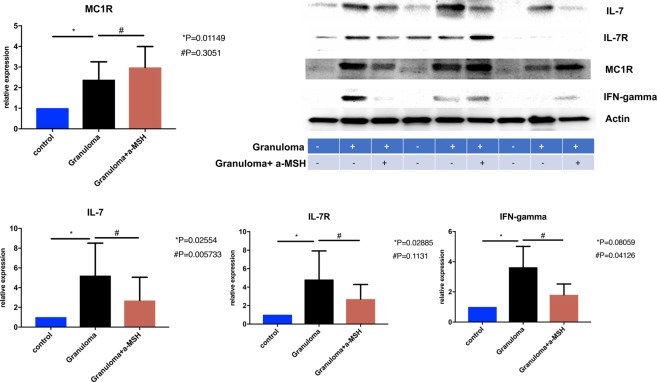
Shows cytokines concentrations of PBMC (control), *in vitro* granulomas (granuloma) and granuloma treated with 𝛼-MSH. Cytokine production of granuloma developed from PBMC of patients with sarcoidosis. Control D0: PBMC without challenging with microparticles in the day of starting experiment. Control D3: PBMC without challenging with microparticles in the day of 3. Granuloma was challenged with 10 μM of 𝛼-MSH.

Protein expression analysis confirmed IL-1b, IL-1R, IL-8, IL-12, CCL3, CCL4, CCL5, GM-CSF, IFN-γ, and TNF-𝛼 were significantly reduced in granulomas treated with 𝛼-MSH (see Fig. 5).

**Figure 6:**
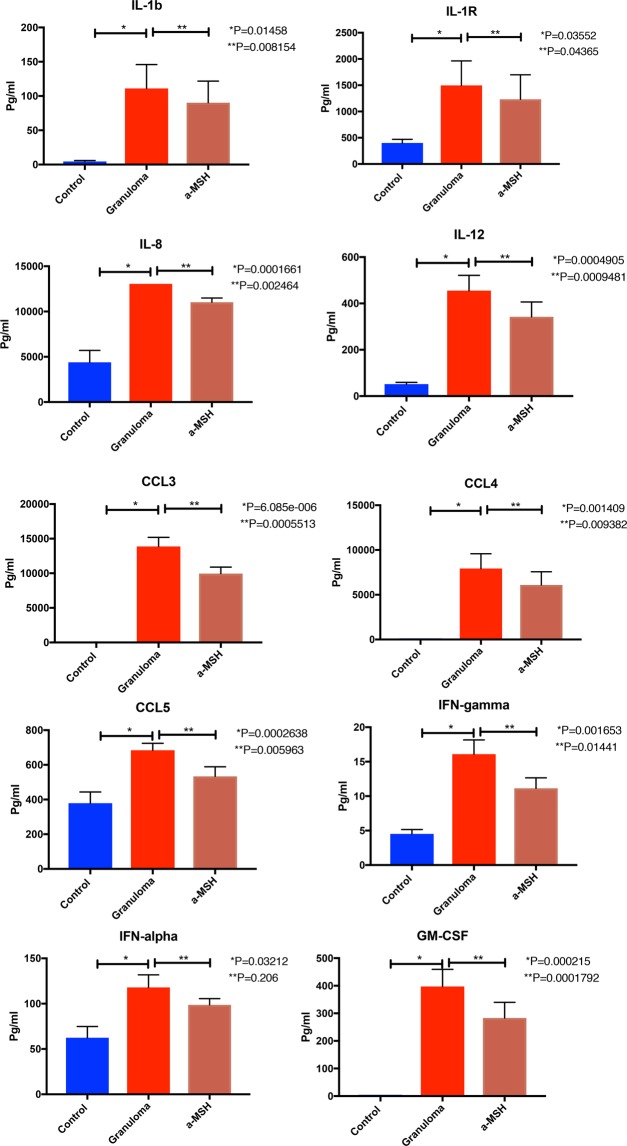
Shows protein expression using western blot in control, granuloma and granuloma treated with α-MSH.

Since 𝛼-MSH belongs to MC1R agonist family, we used an anti-MC1R antibody to assess the western blots in PBMCs, granulomas, and granulomas treated with 𝛼-MSH cells. We found that MC1R expression was significantly increased in the granulomas, but no significant changes occurred after exposure to 𝛼-MSH as shown in Fig. 6.

### α-MSH reduced inflammation in the *in vitro* granulomas by inducing phosphorylation of CREB

Given that p-CREB acts as an important inhibitor of P300/CREB-binding protein coactivator family involved in the transcription of inflammatory mediators^22^, and our observation of p-CREB reduction in granulomas, we hypothesized that induction of p-CREB could be a mechanism of action for 𝛼-MSH. Thus, we measured intracellular p-CREB concentrations in the granulomas treated and untreated with 𝛼-MSHas well as a control group. The data show that the developed granulomas treated with 𝛼-MSH had a significantly increase in the concentration of p-CREB (Fig. 4). Addition of 666–15, a potent and selective CREB inhibitor (IC50 = 81 nM, Sigma-Aldrich, Millipore), significantly blocked the anti-inflammatory effect of 𝛼-MSH (Fig. 4**)**. This strongly suggests that CREB phosphorylation is essential for 𝛼-MSH signaling.

## Discussion

We describe here a novel *in vitro* model to study sarcoidosis. By challenging PBMCs with microparticles generated from MAB cell wall, we were able to induce well-formed granulomas, with clear Th1 and Th17 inflammatory profiles based on gene expression and cytokine secretion studies. We also found that 𝛼-MSH exerts anti-inflammatory effects on this granuloma model with a clear reduction of IL-7, IL-7R, and IFN-*γ*. This therapeutic effect is CREB dependent and reversible using a specific CREB activation inhibitor.

Sarcoidosis causes inflammation in the lungs and triggers a complex cascade of immunopathologic events, including leukocyte recruitment and granuloma formation. Subsequent abnormal repair processes lead to structural changes such as pulmonary remodeling of the lung parenchyma, airway, and vascular systems^23,24^ which result in permanent structural and physiological changes. About a third of patients with sarcoidosis^25^, develop fibrotic pulmonary sarcoidosis that is life-threatening. In sarcoidosis, macrophages and lymphocytes play a crucial role in the inflammatory cascade, both activated by unknown agents on airway epithelium cells and immune cells^26^. The mechanism involved in granuloma formation is not well known. However, it has been suggested that cytokines including IL-2, IL-7, IL-8, IL-12, IFN-*γ*, TNF-*𝛼*, and GM-CSF have an important role in initiation and continuation of granuloma formation. Crouser and co-workers developed an *in vitro* granuloma model using purified protein derivate (PPD) of mycobacterium tuberculosis^27^. They showed that PBMCs react to coated beads with PPD and then aggregate and form granuloma. Our model is more relevant to study sarcoidosis as we have used a more prevalent antigen (NTM is more prevalent than TB and are environmental) and the granulomas are immunophenotypically similar to the granulomas in clinical sarcoidosis. Our generated microparticles developed granuloma in PBMC isolated from all subjects.

We found that the granulomas from sarcoidosis patients had a marked decrease in IFN-γ expression compared to the healthy controls. Richmond *et al*. found the same difference with Th17 cells from sarcoidosis patients^28^. This might be due to reduced antigen recognition by Th17 cells in PBMCs of sarcoidosis patients.

Melanocortin signaling peptides (melanocortins) include adrenocorticotropin hormone (ACTH), α-MSH, beta-melanocyte-stimulating hormone (β-MSH) and gamma-melanocyte-stimulating hormone (γ-MSH). Melanocortins are formed from the POMC prohormone, which is consequently modified by proconvertase 1 or 2^29^. Processing of POMC is specific to the tissues and cells in which it is expressed. Structurally, melanocortins have the amino acid sequence His-Phe-Arg-Trp, which is integral to their biological function^30^. α-MSH is most known for its cutaneous neuroimmunomodulatory response to ultraviolet light that leads to increased skin pigmentation and has further been shown to possess anti-inflammatory^31^ and anti-microbial effects^32^. This is the first report of the anti-inflammatory effect of α-MSH on granulomatous inflammation.

MC1R was originally referred to as α-melanocyte stimulating hormone receptor (the name of its major ligand), though it has equal affinity for ACTH^33^. MC1R is expressed in the immune system, gut, testis, ovary, placenta, lung, liver and skeletal muscle. Specifically, MC1R is found in endothelial cells, monocytes, macrophages, lymphocytes, neutrophils, mast cells, intestinal epithelia among many others^34^. MC1R has also been shown to play a role in inflammation. Certain polymorphisms in MC1R affect the degree of sepsis in patients who have experienced trauma^35^. It has been shown that signaling through MC1R attenuates IL-8 and TNF-α-mediated inflammatory responses^36^. The role of this receptor in pathogenesis of sarcoidosis has not been previously studied.

One of the effects of α-MSH may be reduction in the expression of the MARCO. MARCO is a class A scavenger receptor (SR) that senses and clears pathogens through the recognition of pathogen-associated molecular patterns (PAMPs). MARCO is known for recognizing polyanionic particles in nature, including particulate matters in air, bacterial lipopolysaccharides, DNA, RNA and various intracellular proteins^37^. Many of these ligands are also recognized by Toll-like receptors (TLR) and trigger cell signals through them^38^. Recently, it was demonstrated that TLR-signaling is tightly controlled by MARCO expressed on macrophages^39^. MARCO internalizes antigens to deliver them to TLR3, TLR7/8 and TLR9 which are localized in the cytosol^40^. Although the role of MARCO in sarcoidosis has not been studied, TLRs have a known role in the pathogenesis of sarcoidosis. It was shown that a variant within or close to *TLR4* gene increases susceptibility to sarcoidosis^41^. In animal models, *TLR2* gene deletion was found to be an important factor in the development of granuloma formation. *TLR2* (−/−) mice showed significantly attenuated granuloma inflammation to heat-killed Propionibacterium acnes^42^.

TLR4 is a key receptor for innate immunity against chronic mycobacterial infections^43^. Signaling via TLRs activates NF-κB through phosphorylation and degradation of the inhibitory factor (IkappaB)^44,45^. The observed activation of NF-κB in granuloma could therefore be due to TLR signaling.

We observed that treatment with α-MSH is anti-inflammatory, enhancing NF-κBand p-CREB expression. CREB mediates the transcription of genes containing a cAMP-responsive element. Signaling through G-protein-coupled receptors (GPCRs) induces activation of CREB^46,47^. Given that MC1R is known as GPCR^48^, overexpression of CREB and p-CREB after α-MSH treatment is expected.

CREB signaling is involved in the regulation of cellular proliferation, survival, and differentiation and the transcription of several immune-related genes such as IL-2, IL-6, IL-10 and TNF-alpha as well as macrophage and lymphocytes survival^22,49^. P-CREB is induced in response to cellular stress or growth factors, limits proinflammatory responses by directly inhibiting NF-κBactivation, and blocks binding of NF-κBto CREB binding protein^22^.

Others have shown that α-MSH suppresses NF-κB activation^50^. In our model we found that α-MSH has anti-inflammatory properties by inducing p-CREB despite NF-κB remaining active. The duration of treatment with α-MSH in our model (3 days) vs. short exposure (15 min) in another study may causes the different NF-κB concentration levels.

This study has several limitations. We treated granuloma with 𝛼-MSH for 3 days and could not detect any morphologic changes. The effect of 𝛼-MSH on granuloma size and cell composition should be studied with a longer treatment duration with 𝛼-MSH. The effect of 𝛼-MSH in animal model should be assessed before any consideration for clinical use. We did not measure total CREB and IL10 concentrations in granuloma treated with 𝛼-MSH. The effect of 𝛼-MSH in neutrophils and epithelial cells has not been evaluated yet.

## Conclusion

The current study shows that 𝛼-MSH has anti-inflammatory properties through a significant reduction of IL-7, IL7R, IFN-*γ and CCL3* via downregulation of *MARCO* (gene and protein), and also induction of phosphorylation of CREB. Further investigation is needed to understand the anti-inflammatory effect of 𝛼-MSH in sarcoidosis and explore its potential therapeutic role in sarcoidosis via its anti-inflammatory properties.

## Materials and Methods

Please refer to the online supplement for detailed supplemental images.

### MAB microparticle development

MAB cell wall microparticles were isolated from a strain of MAB with a rough colony isolated from sputum of 11-year old boy with cystic fibrosis (isolate # CCUG 47942, gift from Dr. Malin Ridell, University of Gothenburg, Sweden). MAB was grown in middlebrook 7h9 broth with ADC enrichment medium (Millipore Sigma, St. Louis, MO, USA) at 37 °C. Cells were collected when OD600 reach to 1.0–1.2 by centrifugation at 4000 g for 10 minutes, washed once in PBS, centrifuged and resuspended using a 15 to 1 (volume to volume) ratio of lysis buffer, sonicated and incubated on ice for thirty minutes. The lysis buffer contains 137 mM sodium chloride, 10 mM Na phosphate, 2.7 mM potassium chloride; combined with detergents and protease inhibitors. Lysed cell samples were then centrifuged at 3000 g for 5 minutes to remove intact MAB cells. The supernatant was transferred to a new tube and centrifuged for 20 minutes. 20 ml fresh lysis buffer was and the pellet was resuspended by brief sonication and centrifuged at 12,000 g for another 20 minutes. The pellet was resuspended in 20 ml volume of PBS and kept at 95 °C for 15 min. After cooling to room temperature, the lysate was centrifuged at 12,000 g for 20 minutes and the pellet was washed with PBS buffer 3 times at 12,000 g for 10 minutes. Finally, the pellet was suspended in Dulbecco’s Modified Eagle Medium (DMEM) and stored at −80 °C. The concentration of the microparticles was calculated by the following equation: Final concentration = Volume of original culture x OD600 x (2.2×10^8^ bacteria/ml)/final volume. High quality images of non-infectious, MAB particles were obtained by scanning electron microscope (SEM).

### Human blood sample

Blood samples were collected from 9 patient with confirmed pulmonary sarcoidosis and randomly selected from the University of Miami Sarcoidosis Biobanking, and matched by age, sex and race with 10 healthy control per the University of Miami Institutional Review Board approval no 20150612. To avoid the inconvenience and risks associated with additional venipunctures, a 10 ml blood specimen was collected during an already scheduled venipuncture. Patients who currently had a hgb <7 mg/dL were excluded from participating in this study.

#### PBMC isolation

PBMC were isolated from whole blood using a fully-closed system with Ficoll™ Hypaque™ Solution offered by BD Biosciences, USA (Vacutainer® CPT™ Mononuclear Cell Preparation Tube -Sodium Citrate) per manufacturer recommendation and previous studies protocols^51,52^. All experiments at this study were performed in accordance with relevant guidelines and regulations and approved by Miami VA Healthcare Institutional Review Board (IRB). Informed consent was obtained from all subjects in this study.

#### Cell culture

Isolated PBMC was cultured in RPMI 1640 medium containing 10% autologous serum in 100-by-15-mm or 24-well tissue culture dishes at 37 °C in a 5% CO2 atmosphere. A total of 2 ×10^6^ PBMCs/ml (containing approximately 2 ×10^5^ monocytes), was immediately infected with MAB cell wall microparticles at a MOI of 10:1 in the presence of 10% autologous serum and incubated for up to 7 days, during which time granulomas was developed and studied. Media and serum were replenished every 2 days. Use of autologous serum allows for retention of the undefined characteristics that are unique to each individual. All experiments were repeated 5 times.

### **Bacterial culture and microparticle development**

Briefly, the visible MAB on liquid media were collected, washed with PBS and sonicated in lysis buffer (PBS with 0.1% sds,10 mM EDTA). To remove the intact cells, we pelleted them by centrifuging for 10 minutes at 3000 rpm in tabletop centrifuge. Supernatant was carefully removed and centrifuged again for 10 min at 10,000 rpm to pellet cell wall particles. Pellet was washed one more time with lysis buffer and centrifuged again. Pellet was washed with PBS contain 1% SDS, 10 mM EDTA at heated for 15 min at 95 °C. SDS and EDTA was used to remove contaminating protein and materials ionically-bound to the cell wall. Pellet was Washed with PBS and centrifuging. The microparticles were finally suspend in PBS. Concentrated suspension of microparticles was cultured in The Löwenstein–Jensen medium confirming that intact live bacteria did not pass through the process.

### **Maturing*in-vitro*granuloma like formation**

All experiments in the current study were performed when granuloma reached maturity. The maturity of granuloma formation was determined semi-quantitatively daily (up to 7 days) using the previously described method^53^. As summary, each sample was assessed by light microscopy (Olympus IX71 DP71 microscope digital camera). At least 10 separate high-power fields per sample were evaluated. Matured granuloma was defined if the PBMC after exposure to MAB microparticles formed multilayers, comprising macrophages and lymphocytes cellular differentiation, multinucleated giant cells presence and diameter of the base was in the range of 100 to 200 micrometers.

#### ELISA

Supernatant aliquot samples were analyzed, thawed and spun at 12,000 rpm for 10 min to separate the particulate material at the bottom. 50 μl of undiluted plasma was plated from each sample onto a 96-well V-bottom plate (source plate) by manual pipetting according to predefined maps. The aliquots were wrapped in parafilm and kept in a humid chamber at 4 °C during the entire process, but not longer than 72 hr. Growth factors and their receptor’s capture antibodies were reconstituted and diluted per manufacturer specification and 50 μl plated into each well of respective 96-well high-binding half-well plates which was then sealed and incubated overnight at 4 °C. Alternatively, many plates were dried at 37 °C and stored at 4 °C for later use, depending on the stability of the protein. The ELISA was completed per manufacturer protocol (with volumes adjusted for plates with half-area wells) and the optical density of each well was read using a plate reader set to the appropriate wavelength and analyzed. Cytokines were measured by Human Cytokine Magnetic 25 plex Panel from Life technologies (Carlsbad, CA).

#### Western blot

Total PBMC, granuloma, and granuloma treated with 𝛼-MSH cells’ extracts were prepared using NP-40 lysis buffer (50 mM Tris, PH 8.0, 1.0% NP-40, 150 mM NaCl, 2 mM EGTA, 2 mM EDTA, protease inhibitor tablet (Roche Molecular Biochemicals, Indianapolis, IN), 50 mM Sodium fluoride, and 0.1 mM sodium vanadate), and protein concentrations determined (Bradford method; kit from Pierce). SDS-PAGE separated proteins were electrophoretically transferred to immobilon-P membranes (Millipore Corp. Bedford, MA) and incubated in 5% nonfat dry milk, PBS, and 0.25% Tween-20 for 1 h. Membranes were incubated with primary antibodies overnight at 4 °C, rinsed, incubated with horseradish peroxidase-conjugated secondary antibody, and then exposed to X-ray film (X-Omat, Eastman Kodak Co.) for analysis, using enhanced chemiluminescence (ECL Plus, Amersham Pharmacia Biotech, Arlington, Heights, IL). Actin protein was measured as a loading control. Antibodies used for anti-NF-κB. And anti-p-NF-κB were purchased from cell Signal (Danvers, MA), anti-CREB from Abcam (Cambridge, UK) CREB INHIBITOR, 666–15 from Sigma (St. Louis, MO), anti-IL-7 from Proteintech (Rosemont, IL), CD68, CD4 from Abcam (Cambridge, UK), Anti-MC1 Receptor from Jerusalem, Israel), anti-IL7R Santa Cruz Biotechnology (Dallas, TX), anti-IFN-gamma from Proteintech (Rosemont, IL). More information about antibodies used for this study could be found in supplement 1.

#### Immunofluorescence confocal microscopy

Granuloma was developed in Lab-Tek II Chamber Slide (Thermo Fisher Scientific, Waltham, MA) as previously presented. The PBMC derived from patients were cultured in Chamber Slide treated with microparticle generated from *M. abscessus* cell walls for 72 hours. Removed medium and followed with 3 washes of 1xPBS, and then fixed sample in 4% paraformaldehyde made in PBS for 72 hours. Then washed the slides with 1xPBS 3 times to remove residual paraformaldehyde and permeabilized cells with 0.1% Triton X-100 made in PBS solution for 30 min. Subsequently, 1X Block BSA solution (cat. No. 37520; Thermo Fisher Scientific, Inc.) was used to block the samples for 1 h at room temperature. Samples were treated overnight at 4 °C with the following primary antibodies: CD68 (1:500; cat. no. ab125212; Abcam), PD-L1 (1:200; cat. no. 17952–1-AP; Proteintech Group.), CD4 (1:500; cat. no. ab133616; Abcam). After three rinses in PBS, fluorochrome-conjugated secondary antibody (1:1000 cat. no. 18–0216–32, Rockland, Inc) in 1% BSA/0.05% Triton X were added to the samples for 1 h at room temperature. After 3 times rinses with 1XPBS, slides were counterstained DAPI and sealed. Confocal immunofluorescence images were acquired using a Leica DM6000 microscope with a SP5 confocal module at the University of Miami McKnight Analytical Imaging Core Facility. Captured images were processed using Volocity Software version 6.1.1 software (Perkin-Elmer, Waltham, MA).

#### RNA isolation and analysis

RNA isolation was performed using a kit from ZymoResearch, Irvine, CA USA following the manufacturer recommendations.

### RNA Sequencing at the John Hussman Institute for Human Genomics (HIHG) Center for Genome Technology (CGT)

#### RNA-seq for whole transcriptome analysis

Preparation of transcriptome libraries for sequencing on the Illumina NextSeq. 500 platform was carried out at CGT using established RNA-seq methods. Briefly, 200 ng of total RNA via Agilent Bioanalyzer was prepped for sequencing using a NuGen Universal RNA library preparation kit including AnyDeplete probes to remove ribosomal RNA according to manufacturer’s protocol. Samples were barcoded to allow for multiplexing. Cluster generation and sequencing was taken place on the Illumina NextSeq. 500 using the reagents provided by Illumina, targeting 25 million single-end 75 base reads per sample.

FASTQ files was generated by HiSeq’s Real Time Analysis (RTA) followed by a BCL2FASTQ script supplied by Illumina and then processed through a bioinformatics pipeline. This consists of initial quality control of the reads via FastQC to determine basic quality metrics including per base quality, GC content, and sequence read lengths and distribution. Trimming of the sequences for bad quality bases, adapter and primer sequences were performed using TrimGalore! software. Alignment to the GRCh38 human reference genome was done using STAR v2.5.2a aligner; other methodologies such as HiSat2 or TopHat2 was also considered. The GeneCounts module within STAR was used for gene quantification against the current GENCODE gene definition (currently v28); StringTie and Cufflinks gene quantification were considered as appropriate. Differential expression was performed using the DESeq. 2 software package and pathway analysis based on expression results performed using a variety of programs including DAVID and Ingenuity Pathway Analysis.

The CGT is also equipped with molecular biology (PCR) capabilities, which was used in this study for expression verification and validation. RNASeq analysis isolated from PBMC, granuloma cells, granuloma treated with 𝛼-MSH were analyzed in CGT.

*In vitro* sarcoidosis-like granuloma was developed with challenging PBMC of patients with confirmed sarcoidosis and treatment-naïve with microparticles generated from *Mycobacterium abscessus* cell wall. PBMC, and developed granuloma were treated daily with 1 μM and 10 μM of 𝛼-MSH (MILLIPORE-SIGMA, St. Louis, MO, USA) or saline as control. The supernatants of developed *in vitro* granuloma treated with saline and 𝛼-MSH were collected on Day 3. The cytokine concentrations were measured using Illumina multiplex Elisa as mentioned previously. The PBMC, granuloma and granuloma + 𝛼-MSH cells from were harvested and cellular protein was extracted for western blotting as previously described. To determine the anti-inflammatory effects of 𝛼-MSH on granuloma protein and gene expressions of IL-7, IL-7R, IFN-*γ*, MC1R, NF-κB, and p-NF-κB, MARCO, and p-CREB were measured with western blot and RNAseq respectively. and stored *frozen* inside a −80 freezer no longer a year after isolation.

#### RT-PCR

RT-PCR was performed as previously described. MARCO, IL-6, IL-7. IL-8, IL17A, IFN-gamma were analyzed. The list of primers can fine in supplement 1.

## Supplementary information

Supplental document.
