# Supplementary material for: Anti-inflammatory effects of α-MSH through p-CREB expression in sarcoidosis like granuloma model
Source: Sci Rep. 2020 Apr 29;10:7277. doi: 10.1038/s41598-020-64305-9 (PMC7190699; doi:10.1038/s41598-020-64305-9)
Supplement: Supplementary file 1 — Supplental document. [file 41598_2020_64305_MOESM1_ESM.pdf]

Anti-inflammatory effects of α-MSH through p-CREB expression in sarcoidosis like granuloma model

Chongxu Zhang PhD[1], Stephanie Chery MD[2], Aaron Lazerson DVM[3], Norman H Altman DVM[3], Robert Jackson MD[1,4], Greg Holt MD[1,4], Michael Campos MD[1,4], Andrew V Schally PhD[5], and Mehdi Mirsaeidi MD[1,4]

[1] Section of Pulmonary, Miami VA Health System, Miami, FL, [2]Departments of Medicine University of Miami Miller School of Medicine, Miami, FL, [3]Comparative Pathology, University of Miami Miller School of Medicine, Miami, FL, [4]Division of Pulmonary and Critical Care, University of Miami Miller School of Medicine, Miami, FL, [5]Polypeptide and Cancer Institute, Veterans Affairs Medical Center, Miami, FL

Correspondence author:

Dr. Mehdi Mirsaeidi

Division of Pulmonary and Critical Care, University of Miami, Miami, FL, USA.
Email: msm249@med.miami.edu.

Supplement document

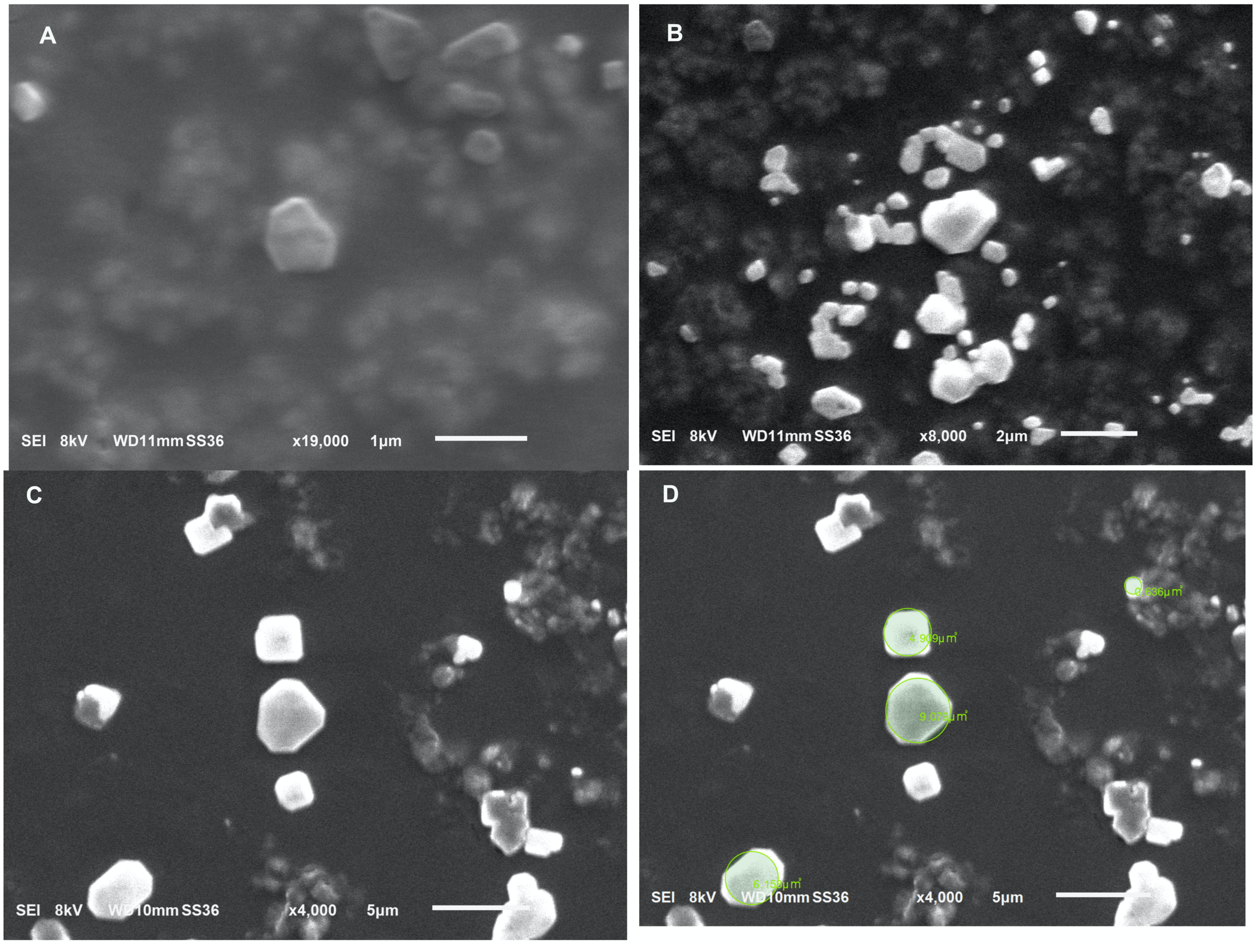

Figure S1. Shows SEM images from microparticles from MAB. A, B, C) shows cluster of particles with scale bar of 1, 2, and μm respectively. D) shows surface area of particles ranging from <1 μm to 9 μm.

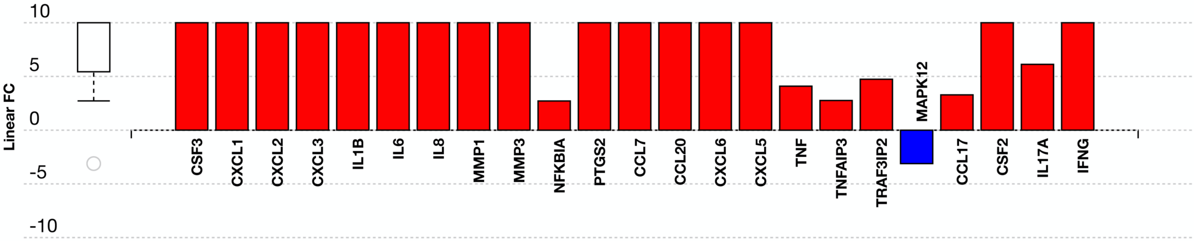

Figure S2. Shows analysis of RNASeq from granuloma developed from PBMC of sarcoidosis subjects treated with microparticles vs. PBMC of the same patients treated saline. The results show that developed granuloma overexpressed several immune genes including *IL8*, *IL6*, *TNF-a*, *IL-17a*, *IFN-gamma*, *CCL7* and *CSF3*. (Corrected P value (FDR) <0.01, and Fold Change 2.5). *CSF3*: Colony Stimulating Factor 3, *CXCL1*: C-X-C Motif Chemokine Ligand 1, *CXCL2*: C-X-C Motif Chemokine Ligand 2, *CXCL3*: C-X-C Motif Chemokine Ligand 3, *CXCL5*: C-X-C Motif Chemokine Ligand 5, *CXCL6*: C-X-C Motif Chemokine Ligand 6, *IL1B*: Interleukin 1 Beta, *IL6*: Interleukin 6, *IL8*: Interleukin 8, *MMP1:* Matrix Metallopeptidase 1, *MMP3:* Matrix Metallopeptidase 3, *NFKBIA*: NFKB Inhibitor Alpha, *PTGS2:* Prostaglandin-Endoperoxide Synthase 2, *CCL7*: Chemokine (C-C motif) ligand 7, *CCL20*: Chemokine (C-C motif) ligand 20, *TNF:* Tumor necrosis factor family, *TNFAIP2*: TNF Alpha Induced Protein 2, *MAPK12*: Mitogen-Activated Protein Kinase 12, CCL17: *CCL7*: Chemokine (C-C motif) ligand 17, *CSF2*: Colony Stimulating Factor 2, *IL-17A:* Interleukin 17A, *IFNG*: Interferon gamma

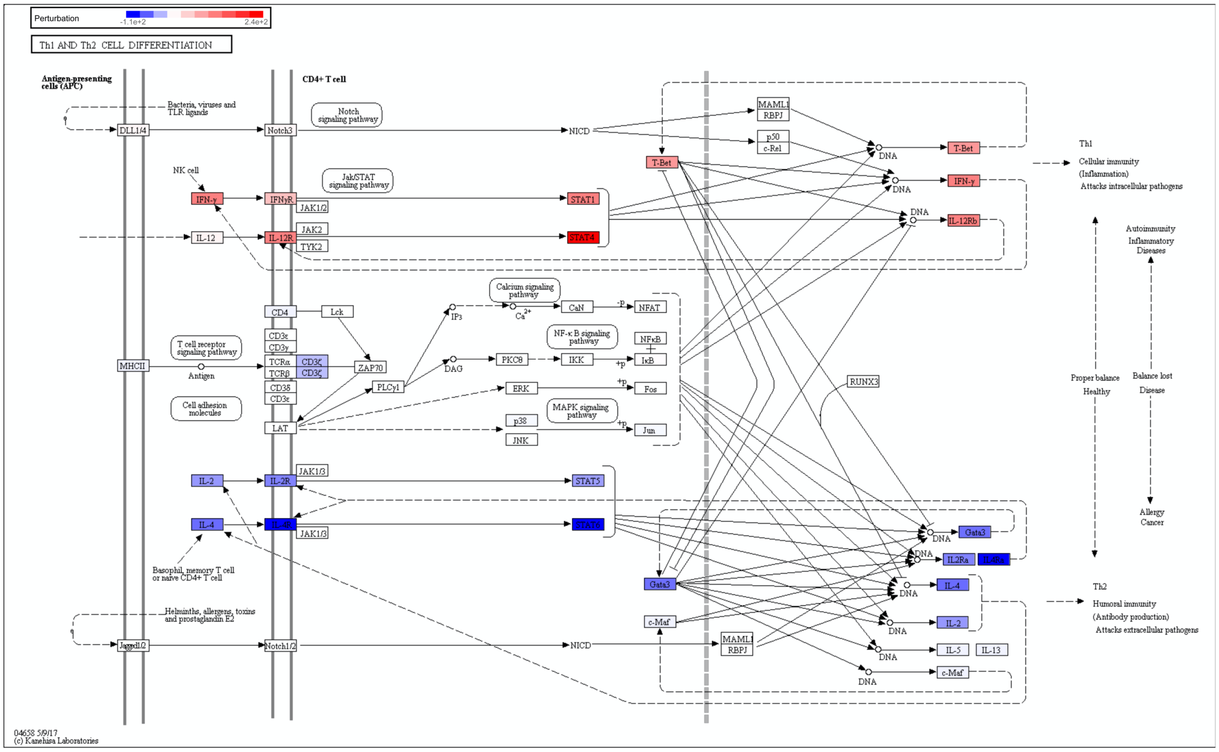

Figure S3. Shows pathway analysis of RNASeq from granuloma developed from PBMC of sarcoidosis subjects treated with microparticles vs. PBMC of the same patients treated saline. The results show that the developed granuloma overexpressed several immune genes in Th-1 pathway. *IFN-gamma* and *IFN-gamma R*, *IL-12R*, *STAT 1* and *STAT 4*, and *T Bet* are overexpressed, but *IL-4* and *IL-4 R*, *STAT 5* and *STAT 6*, were downregulated. (Corrected P value (FDR) <0.01, and Fold Change 2.5). Copyright permission of KEGG pathway maps was acquired from the Kanehisa laboratory.

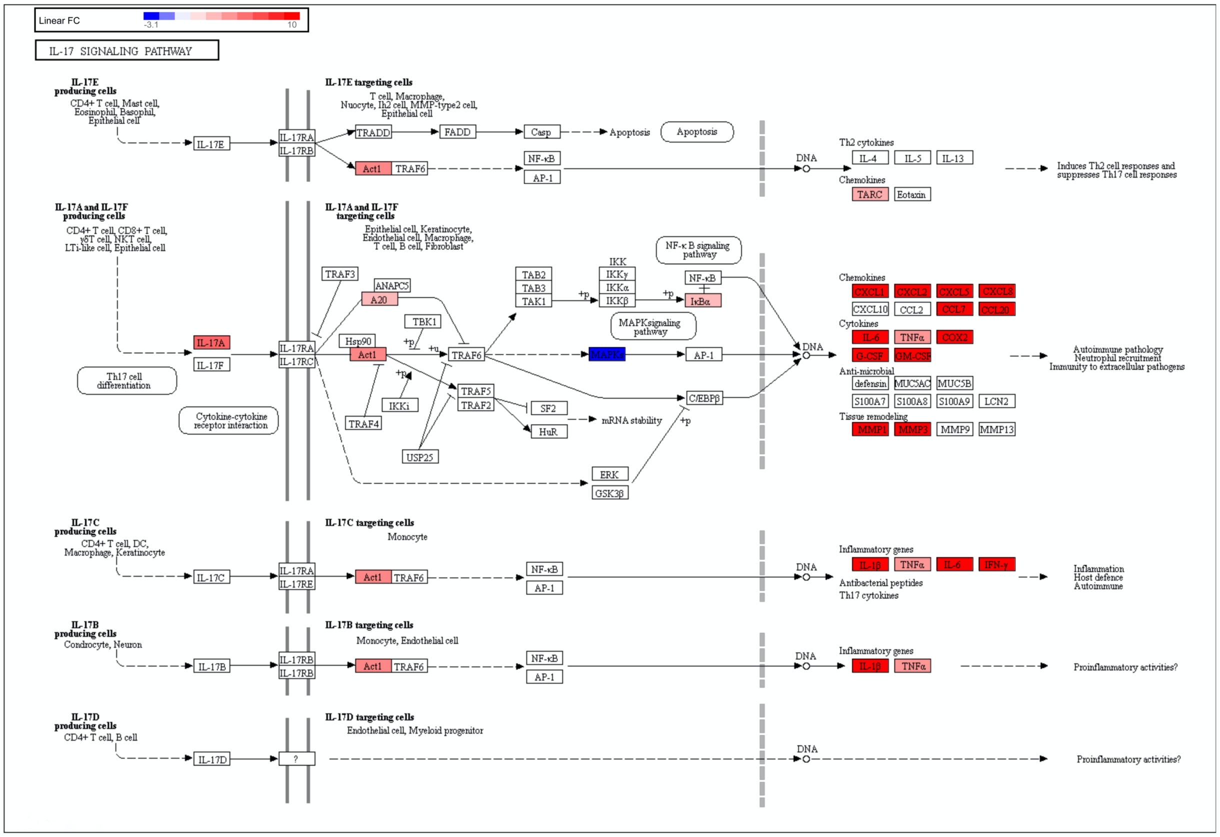

Figure S4. Shows pathway analysis of RNASeq from granuloma developed from PBMC of sarcoidosis subjects treated with microparticles vs. PBMC of the same patients treated saline. The results show that the developed granuloma overexpressed several immune genes in *IL-17* pathway. *IL-17A*, *Act1*, and *A20* overexpressed, but *MAPK* downregulated. (Corrected P value (FDR) <0.01, and Fold Change 2.5). Copyright permission of KEGG pathway maps was acquired from the Kanehisa laboratory.

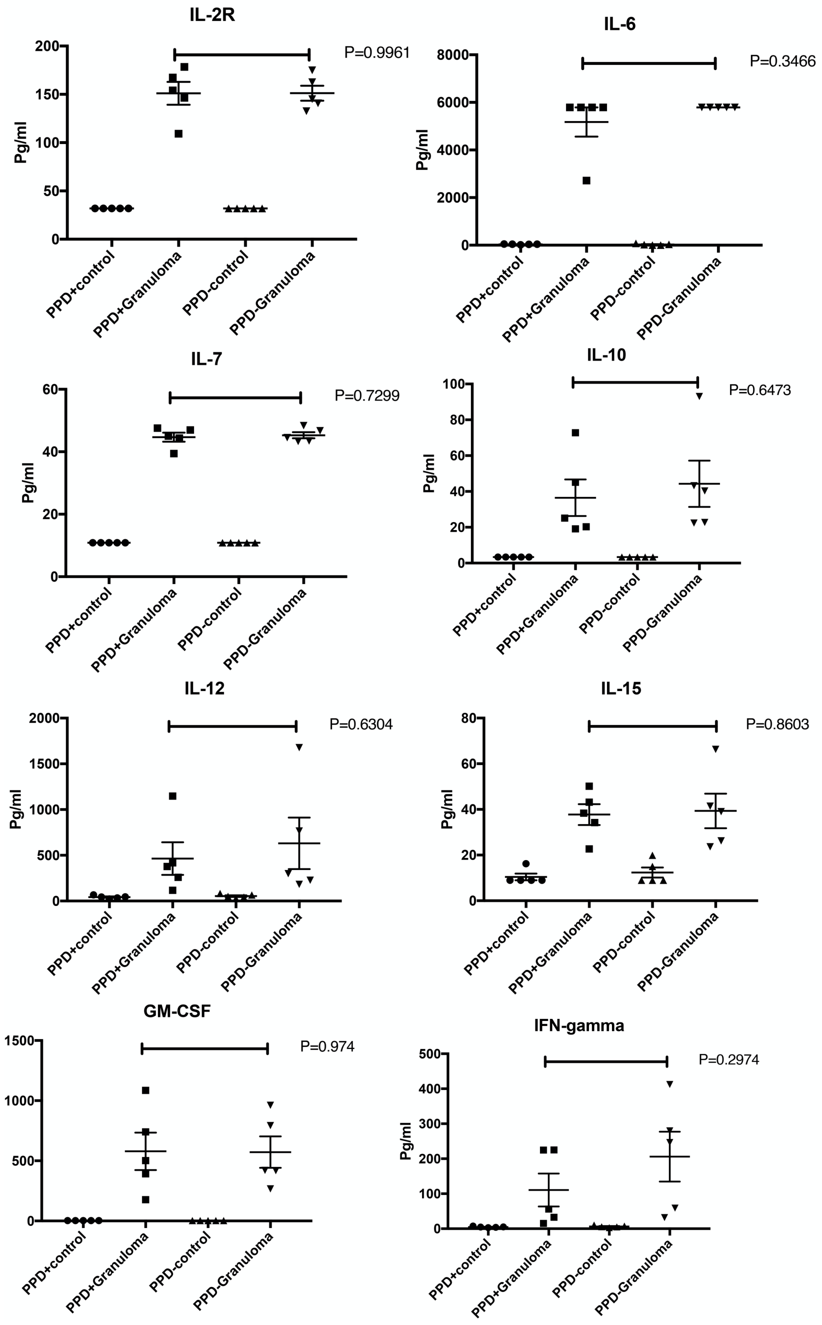
IL-2R
P=0.9961
IL-6
P=0.3466
IL-7
P=0.7299
IL-10
P=0.6473
IL-12
P=0.6304
IL-15
P=0.8603
GM-CSF
P=0.974
IFN-gamma
P=0.2974
Pg/ml
PPD+control
PPD+Granuloma
PPD-control
PPD-Granuloma

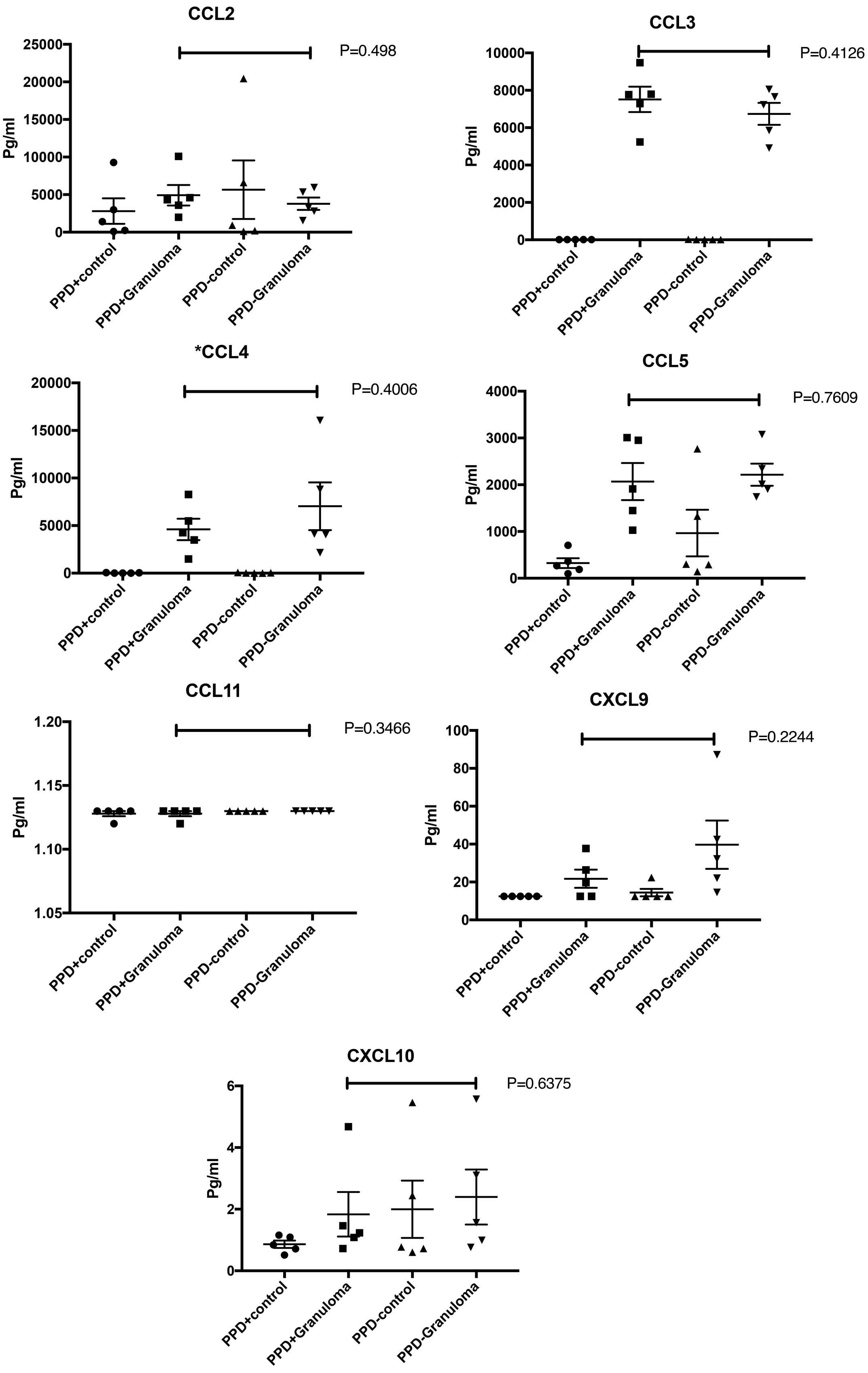
CCL2
P=0.498
Pg/ml
25000
20000
15000
10000
5000
0
PPD+control
PPD+Granuloma
PPD-control
PPD-Granuloma
CCL3
P=0.4126
Pg/ml
10000
8000
6000
4000
2000
0
PPD+control
PPD+Granuloma
PPD-control
PPD-Granuloma
*CCL4
P=0.4006
Pg/ml
20000
15000
10000
5000
0
PPD+control
PPD+Granuloma
PPD-control
PPD-Granuloma
CCL5
P=0.7609
Pg/ml
4000
3000
2000
1000
0
PPD+control
PPD+Granuloma
PPD-control
PPD-Granuloma
CCL11
P=0.3466
Pg/ml
1.20
1.15
1.10
1.05
PPD+control
PPD+Granuloma
PPD-control
PPD-Granuloma
CXCL9
P=0.2244
Pg/ml
100
80
60
40
20
0
PPD+control
PPD+Granuloma
PPD-control
PPD-Granuloma
CXCL10
P=0.6375
Pg/ml
6
4
2
0
PPD+control
PPD+Granuloma
PPD-control
PPD-Granuloma

Figure S5. Shows cytokine profiles of PBMC from healthy subjects with and without a positive PPD

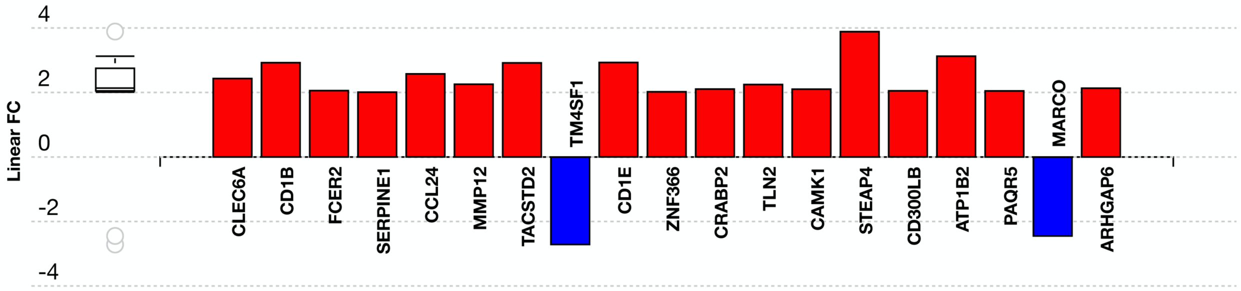

Figure S6. Shows pathway analysis of RNASeq in comparison between granuloma developed from PBMC of sarcoidosis subjects treated with α-MSH vs. granuloma developed from PBMC of sarcoidosis subjects with microparticles but treated with saline *MARCO* was down-expressed with alpha-MSH in granuloma. (Corrected P value (FDR) <0.01, and Fold Change 2). *CLEC6A*: C-Type Lectin Domain Containing 6A, *CD1B*: cluster of differentiation 1, *FCER2*: Fc epsilon RII, *SERPINE1*: Serpin Family E Member 1, *CCL24*: Chemokine (C-C motif) ligand 24, *MMP12*: Matrix Metalloproteinase 12 (Macrophage Elastase), *TACSTD2*: Tumor Associated Calcium Signal Transducer 2, *TM4SF1:* Transmembrane 4 L6 family member 1, *CD1E*: cluster of differentiation 1 E (T-cell surface glycoprotein), *ZNF366*: Zinc finger protein 366, also known as DC-SCRIPT (Dendritic cell-specific transcript), *CRABP2*: Cellular retinoic acid-binding protein 2, *TLN2*: Talin 2, *CAMK1*: Calcium/calmodulin-dependent protein kinase type 1, *STEAP4: STEAP4* Metalloreductase, *CD300LB*: CD300 Molecule Like Family Member B, *ATP1B2*: ATPase, Na+/K+ transporting, beta 2, *PAQR5:* Progestin And AdipoQ Receptor Family Member 5, *MARCO*: Macrophage Receptor With Collagenous Structure, *ARHGAP6*: Rho GTPase Activating Protein 6

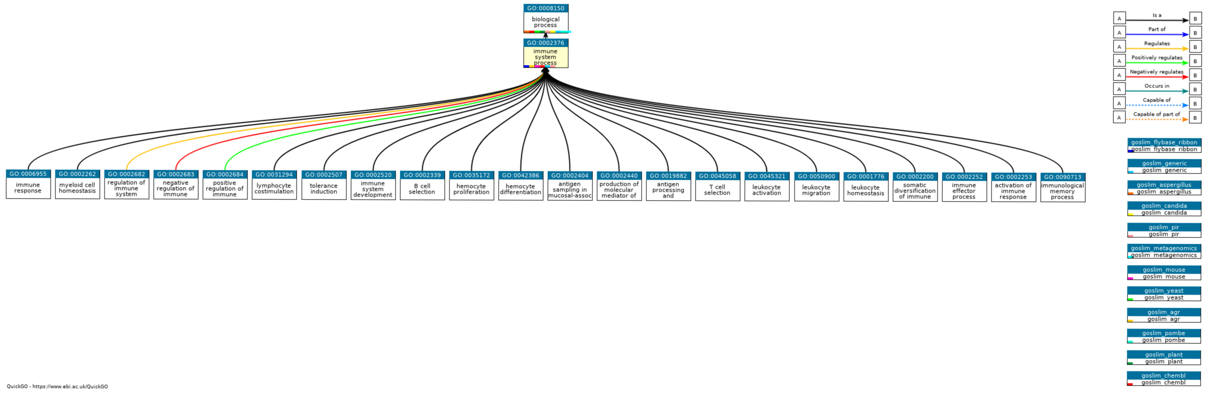

Figure S7. Shows biologic process of RNASeq from granuloma developed from PBMC of sarcoidosis subjects treated with α-MSH vs. granuloma of the same patients treated saline. DAVID version 6.8 was used to perform analysis. The results show treatment with a-MSH activates pathways related to immune system process including tolerance induction. (Corrected P value (FDR) <0.05, and Fold Change 2.5)

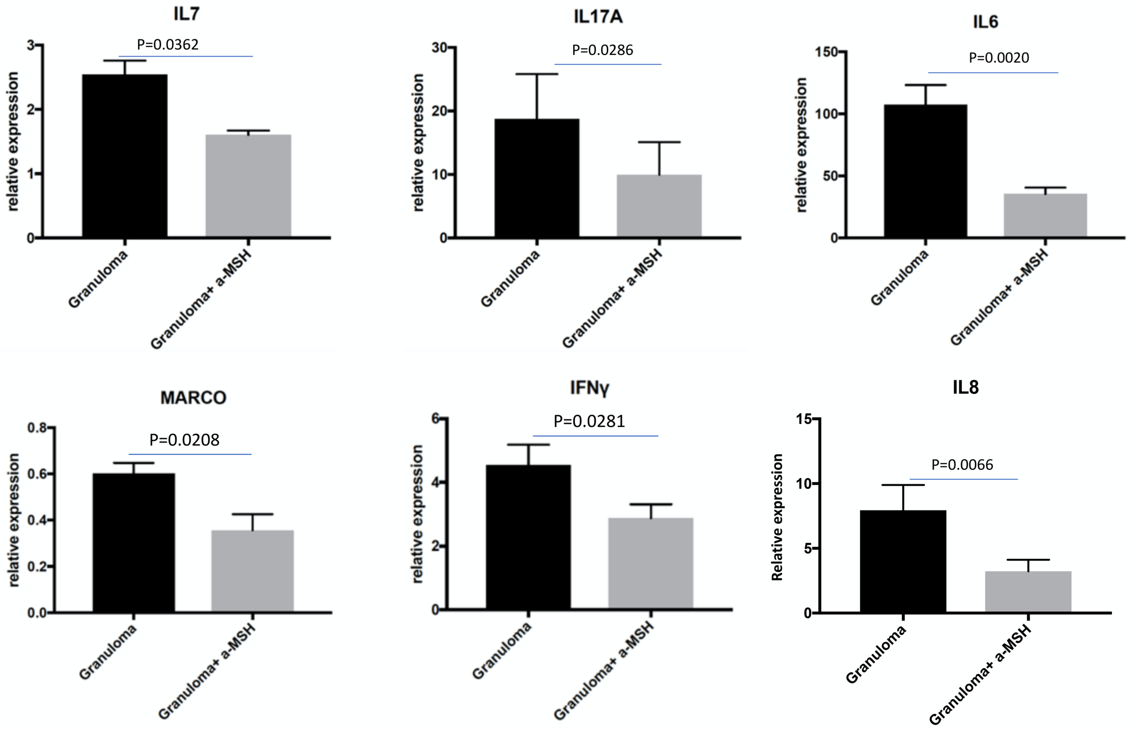

Figure S8. shows gene expression profiles of developed granuloma from PBMC of subjects with sarcoidosis and treated with a-MSH using RT-PCR.

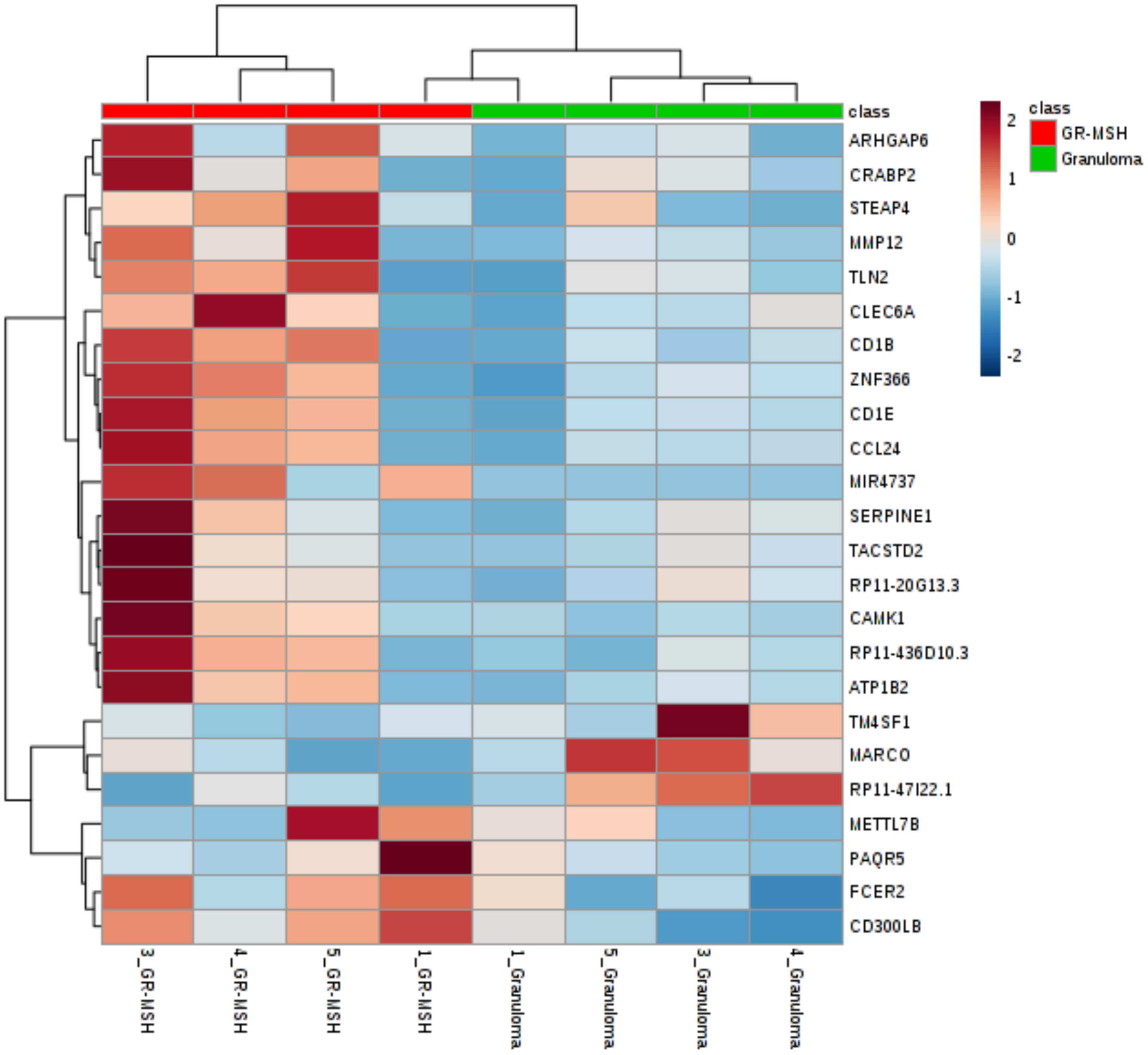

Figure S9. Heatmap for genes in comparison between granuloma developed from PBMC of sarcoidosis subjects treated with alpha-MSH vs. granuloma developed from PBMC of sarcoidosis subjects with microparticles but treated with saline (Corrected P value (FDR) <0.01, and Fold Change 2). Granuloma: developed from PBMC of sarcoidosis subjects with microparticles treated with saline, GR-MSH: granuloma developed from PBMC of sarcoidosis subjects treated with alpha-MSH
